# Spinal Cord Subacute Combined Degeneration Mimicked by Copper Deficiency: A Case Report

**DOI:** 10.1002/ccr3.70190

**Published:** 2025-02-07

**Authors:** Zekarias Seifu Ayalew, Mehariw Wondimu Netsere, Matyas Adugna Abebe, Surafel Tilahun Maru, Gebeyehu Tessema Azibte, Aynalem Demsis Biza, Getnet Yigzaw Mossie

**Affiliations:** ^1^ Department of Internal Medicine Addis Ababa University Addis Ababa Ethiopia; ^2^ Department of Internal Medicine Saint Paul's Hospital Millennium Medical College Addis Ababa Ethiopia; ^3^ Department of Neurology Bahirdar University Bahirdar Ethiopia; ^4^ Department of Neurology Addis Ababa University Addis Ababa Ethiopia; ^5^ Bethel Medical College Addis Ababa Ethiopia

**Keywords:** dorsal column dysfunction, myelopathy, paraplegia, sensory ataxia, vitamin B12 deficiency

## Abstract

This case report underscores the diagnostic challenge of copper‐deficiency myelopathy (CDM) and subacute combined degeneration (SCD) due to their similar clinical presentations. A 32‐year‐old male farmer initially treated for SCD with normal vitamin B12 levels showed no improvement, leading to a delayed diagnosis of CDM. His symptoms included sensory ataxia, spasticity, and sensory loss in the lower extremities, which resolved with oral copper supplementation. Clinicians should maintain a high index of suspicion for CDM, especially in patients who are unresponsive to B12 therapy. This case also highlights that marginal copper deficiency can cause significant neurological symptoms and that early intervention with copper supplementation can lead to full recovery, preventing irreversible damage. It also emphasizes the need for comprehensive testing, including copper levels, in cases of atypical myelopathy to avoid a delayed diagnosis.

**Abbreviations**
ANA
: antinuclear antibody
B12
: vitamin B12
CSF
: cerebrospinal fluid
HIV
: human immunodeficiency virus
HTLV1
: human T‐cell leukemia virus type 1
MRI
: magnetic resonance imaging
RBC
: red blood cell
SCD
: subacute combined degeneration
TTG
: tissue transglutaminase
VDRL
: Venereal Disease Research Laboratory

## 1 Introduction

Copper‐deficiency myelopathy (CDM) is a rare condition characterized by progressive dysfunction of the dorsal columns, which leads to sensory ataxia, weakness, and spasticity [1]. Copper is an essential trace element that serves as a cofactor for numerous enzymes critical to the nervous system's structure and function, including those involved in mitochondrial energy production, iron metabolism, and antioxidant defense [2, 3].

The primary cause of copper deficiency is associated with reduced absorption in the stomach and proximal duodenum. This condition is frequently associated with upper gastrointestinal surgery, malabsorption syndromes such as celiac disease, and excessive intake of exogenous zinc [4]. In instances of excessive zinc intake, zinc disrupts the intestinal absorption of copper by upregulating a ligand that preferentially binds to and sequesters copper within enterocytes [5, 6, 7].

Dietary copper deficiency is uncommon, but it has been documented in premature infants fed milk formulas lacking sufficient copper supplementation [8, 9]. Additionally, copper deficiency has been observed in at least one patient with alcohol use disorder [10].

The clinical and radiological presentation of CDM often closely resembles subacute combined degeneration (SCD) caused by vitamin B12 (cobalamin) deficiency [11, 12]. Other less frequently reported and less clearly causally related neurological conditions associated with acquired copper deficiency include isolated peripheral neuropathy, motor neuron disease, myopathy, cerebral demyelination, cognitive dysfunction, and optic neuropathy [13, 14, 15, 16, 17, 18, 19, 20].

Patients typically present with gait difficulties, primarily due to sensory ataxia from dorsal column dysfunction and, to a lesser extent, spasticity. Paresthesia of the upper and lower limbs is common, whereas urinary symptoms are uncommon. On examinations, spastic paraparesis or tetraparesis with a truncal sensory level for the dorsal column modalities is usually observed. Additionally, sensory/motor neuropathy often coexists, presenting as depressed distal reflexes and superimposed sensory impairment in a glove and stocking distribution [21].

It has been proposed that CDM may result from the dysfunction of cytochrome‐c oxidase, an enzyme that relies on copper [4]. The methylation cycle dysfunction is considered the initial step in a shared final pathway of CDM and SCD [4]. SCD is attributed to the dysfunction of methionine synthase, a crucial component of the cycle. Both methionine synthase and S‐adenosylhomocysteine hydrolase may rely on copper. This hypothesis could explain the clinical and radiological similarities between CDM and SCD, though direct biochemical evidence is still needed to confirm this theory.

CDM is often caused by significantly reduced serum copper levels, with roughly 50% of cases showing levels below 0.1 μg/mL [21].

CDM is almost always caused by poor copper absorption in the gut. However, oral copper supplementation is an effective and suitable treatment option. Sometimes, patients receive copper intravenously first to quickly restore copper levels and prevent further decline before switching to oral maintenance therapy [21]. Herein, we report the case of a 32‐year‐old male who presented with the typical clinical manifestations of SCD and was treated for SCD without improvement, leading to a delayed diagnosis of CDM. This case highlights the importance of a high clinical suspicion of CDM in patients presenting with SCD.

## 2 Case History/Examination

A 32‐year‐old man presented with numbness, tingling, and weakness in his legs for 2 months. Before the onset of his symptoms, he complained of sudden vibrations that began in his left lower extremity, gradually increased in frequency, and became bilateral. These sensations would come and go at first but eventually become more persistent. He then experienced numbness and tingling over his bilateral toes and electric shock‐like sensations on the posterior side of the neck with movement but no radiation to the extremities. At first, he struggled to walk briskly and later found it difficult to keep up at his usual pace. Over 2 months, his condition progressively worsened, requiring him to use a wheelchair for mobility. The patient did not report any exacerbating or relieving symptoms. He denied any past medical or psychiatric illnesses and reported no family history of similar conditions. Otherwise, he did not have any preceding illness, surgery, or trauma to his back. He had no history of skin rash, fever, joint pain, dryness of the mouth, headaches, speech or visual complaints, or bowel or bladder control problems.

The patient was a married farmer who lived with his wife and children. Regarding dietary history, the patient usually eats locally prepared injera (a thin bread made of teff, an Ethiopian staple food) with a stew made of beans, peppers, onions, and black pepper, and occasionally meat, dairy products, and fruits, such as bananas, oranges, and watermelons.

Physical examination reveals blood pressure of 100/60 mmHg, pulse rate of 86 beats per minute, respiratory rate of 16 breaths per minute, temperature of 36.5°C, oxygen saturation of 94%, and unremarkable general physical examinations. He was alert and oriented to person, place, and time. No cranial nerve abnormalities were observed. His upper extremities were unaffected. Bilateral muscle power was assessed for hip flexors, hip extensors, hip abductors, hip adductors, knee flexors, knee extensors, ankle dorsiflexors, and ankle plantar flexors. All muscle groups were graded 3/5. Increased deep tendon reflexes in the lower extremities, including brisk patellar and ankle clonus, were present bilaterally, and a positive Babinski sign was also noted. Notably, bilateral lower extremity vibration and position senses were absent in the toes but intact elsewhere, whereas pinprick and temperature sensations remained normal.

### 2.1 Methods

**FIGURE 1:**
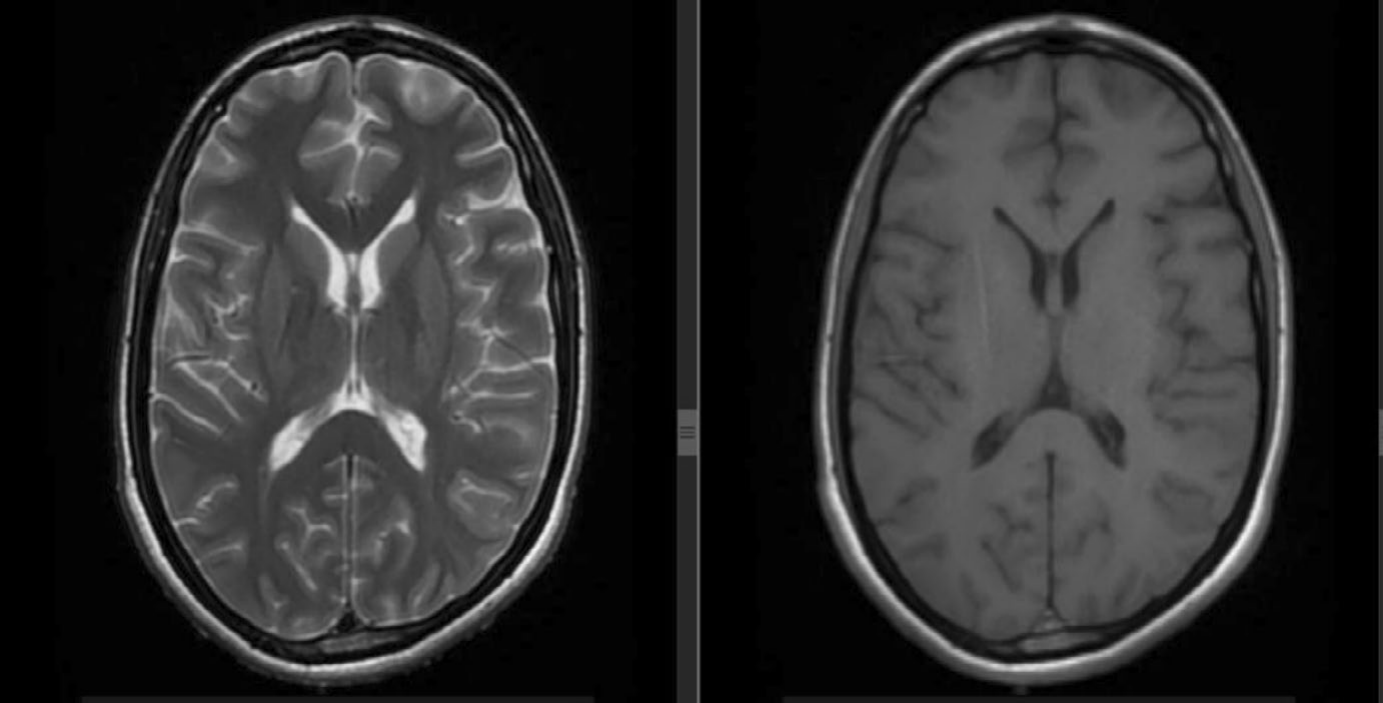
T1 weighted (right) and T2 weighted (left) brain MRI (normal). MRI, magnetic resonance imaging.

**FIGURE 2:**
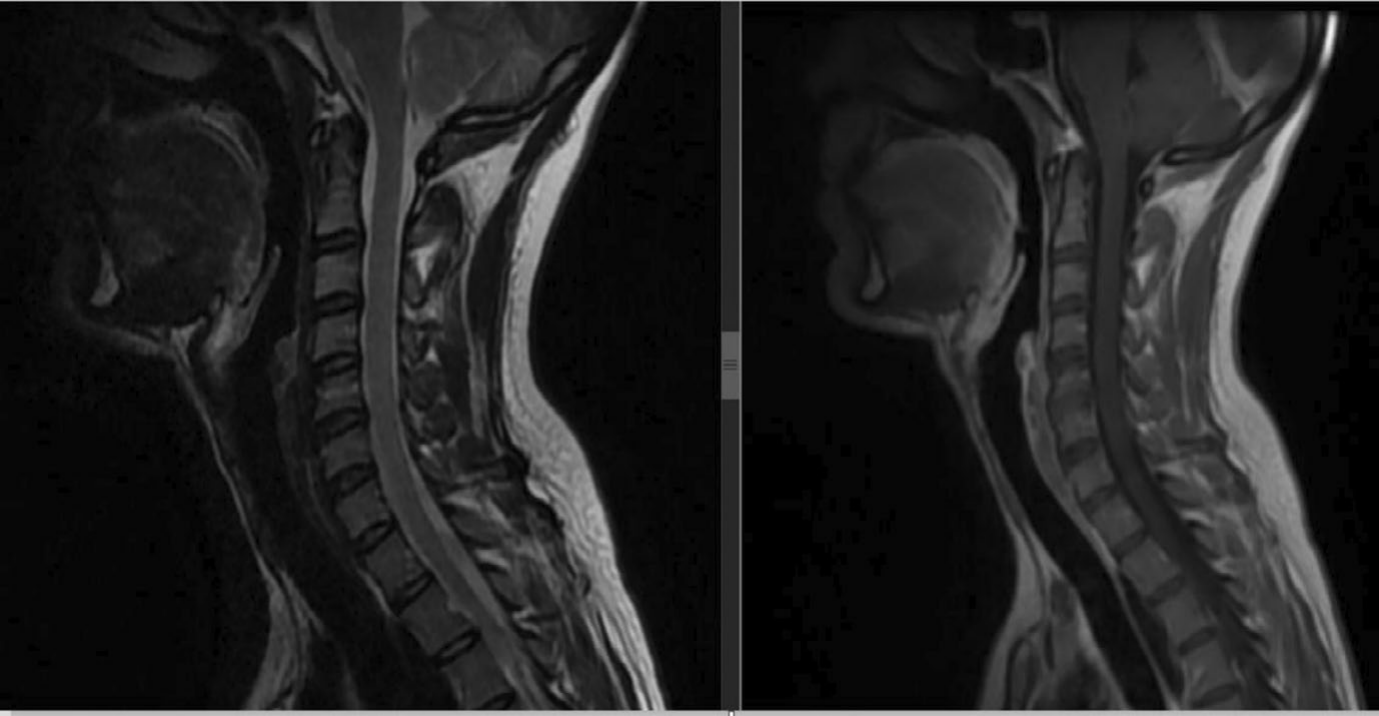
T1 weighted (right) and T2 weighted (left) cervical MRI sagittal view (normal). MRI, magnetic resonance imaging.

**FIGURE 3:**
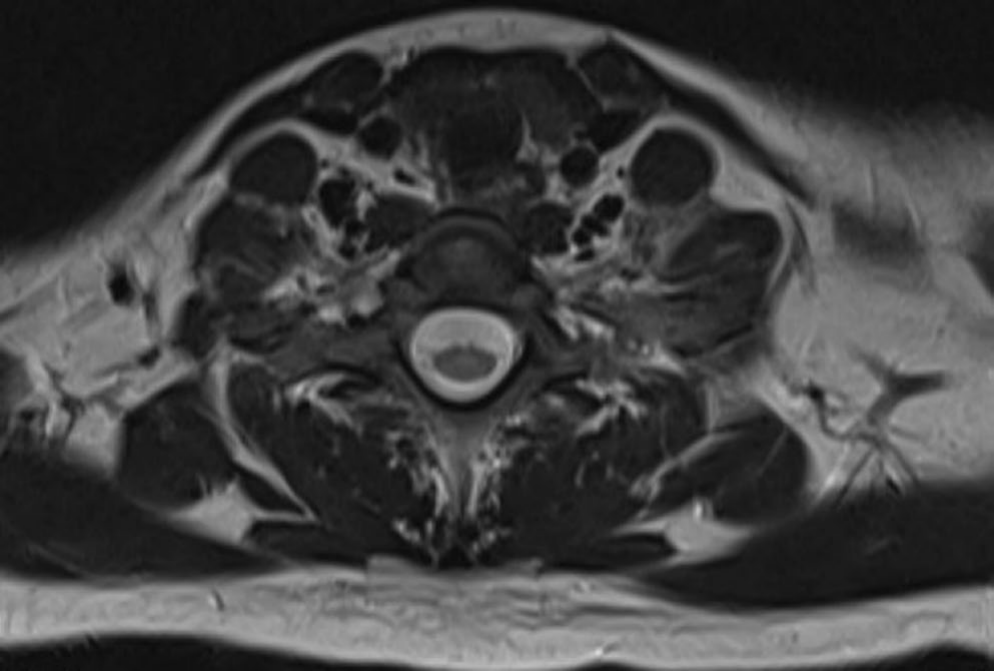
Cervical spine MRI T2‐weighted axial view at C6‐C7 level (normal) MRI, magnetic resonance imaging.

**FIGURE 4:**
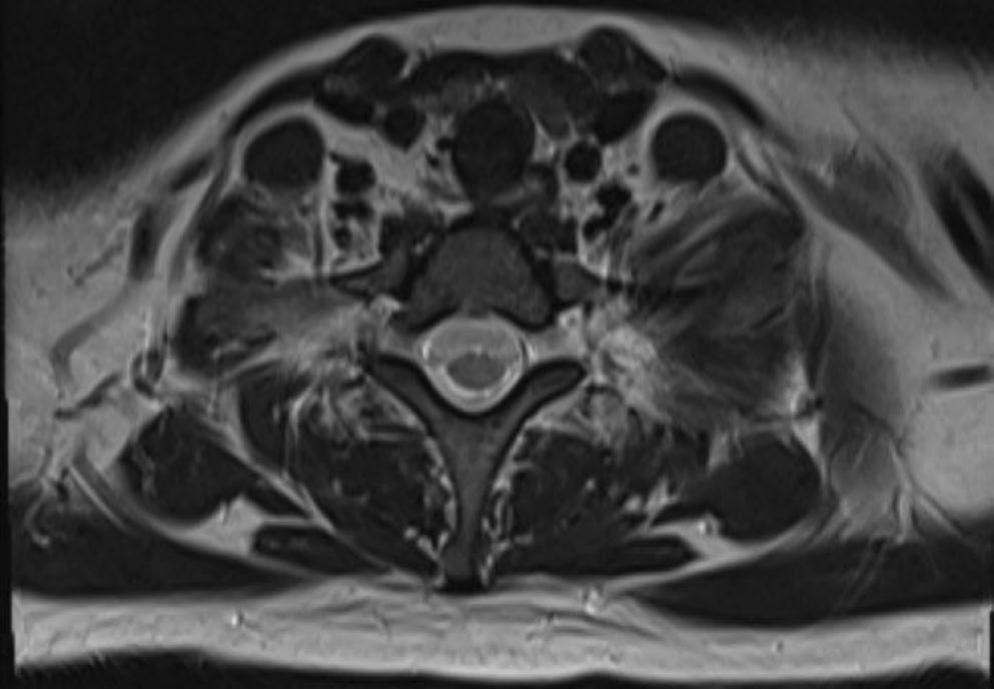
Cervical spine MRI T2‐weighted axial view at C7‐T1 level (normal).

The complete blood count and RBC indices were within normal range, and CRP and ESR were 0.8 mg/dL and 5 mm/h, respectively. Organ function and thyroid function test results were all within normal ranges. Cerebrospinal fluid analysis (cell count, protein, VDRL, aquaporin‐4 antibody), viral markers (Hepatitis B and C, HIV), and antinuclear antibody (ANA) testing showed no abnormalities, which makes alternative infectious and autoimmune diagnosis less likely (Table 1). Brain and spinal cord MRI (Figures 1, 2, 3, 4) were unremarkable. Serum vitamin B12 level was 416 pg/mL (reference range: 200–900 pg/mL). The serum copper level was low at 63.03 μmol/L (reference range: 70–140 μmol/L) (Table 1). A nerve conduction test was performed, and the results were unremarkable. Electrophysiological studies were not performed because they were not available at our institution. Malabsorption evaluations were all negative, including serum tissue transglutaminase (TTG) antibody, colonoscopy with biopsy, and fecal calprotectin tests.

**TABLE 1:** Laboratory profile of the patient with normal reference ranges.

Laboratory	Value	Reference range
WBC	5600 cells/μL	5000–11,000 cells/μL
Hemoglobin	13.9 mg/dL	13–16 mg/dL
Platelets	2,650,000 cells/μL	150,000–450,000 cells/μL
Creatinine	0.7 mg/dL	0.8–1.1 mg/dL
BUN	8 mg/dL	5–20 mg/dL
AST	20 IU/L	5–35 IU/L
ALT	18 IU/L	5–35 IU/L
ALP	120 IU/L	50–180 IU/L
FBS	90 mg/dL	70–100 mg/dL
Serum sodium	140 mEq/L	135–145 mEq/L
Serum potassium	3.6 mEq/L	3.5–5.5 mEq/L
Serum magnesium	1.8 mg/dL	1.6–2.5 mg/dL
Serum calcium	8.6 mg/dL	8–10 mg/dL
Serum chloride	100 mEq/L	95–105 mEq/L
Serum phosphate	3.6 mg/dL	3.4–4.5 mg/dL
Serum vitamin B12	416 pg/mL	200–900 pg/mL
Serum TSH	1.2 IU/mL	0.3–5.6 IU/mL
Serum FT4	14.1 ng/dL	0.6–22 ng/dL
Serum FT3	2.4 pg/mL)	2.3–6.7 pg/mL
Serum copper	63.03 μmol/L	70–140 μmol/L
HBSAG	Negative	Negative
HCV‐Antibody	Negative	Negative
HIV test	Negative	Negative
VDRL	Negative	Negative

Abbreviations: ALP, alkaline phosphatase‐fasting blood sugar; ALT, alanine transaminase; AST, aspartate transaminase; BUN, blood urea nitrogen; FT3, free triiodothyronine; FT4, free thyroxine; HBSAG, hepatitis B surface antigen; HCV, hepatitis C virus; TSH, thyroid stimulating hormone; VDRL, venereal disease research laboratory; WBC, white blood cells.

## 3 Results and Conclusion

Initially, the patient received empirical treatment for suspected vitamin B12 deficiency despite his normal serum vitamin B12 levels (cyanocobalamin 1 mg intramuscularly per day for 1 week, then 1 mg intramuscularly weekly for a month). However, the patient did not show any improvement. Copper supplementation with 2 mg of cupric chloride (equivalent to 2 mg of elemental copper) was initiated orally once daily. His serum copper level normalized, and he was treated for 9 months. Three months after starting treatment, his serum copper level was 72 μmol/L (reference range, 70–140 μmol/L). He began using a walker in the second month of therapy and progressed to walking with bilateral crutches in the third month. By the fourth month, he was able to use only one crutch. By the sixth month, he had achieved unsupported walking. After 8 months of treatment, the patient returned to work.

As the initial MRI and nerve conduction tests were unremarkable, repeat imaging and nerve conduction studies were not performed at the end of the follow‐up, and complete blood count, organ function tests, and serum electrolytes were normal. This case highlights the importance of CDM in the differential diagnosis of SCD. A patient with SCD‐like symptoms who was unresponsive to B12 therapy was diagnosed with CDM based on a low serum copper level. Oral copper supplementation led to complete neurological recovery.

This case also demonstrates that significant neurological impairment can result from marginal copper deficiency and emphasizes the need for high clinical suspicion of CDM to ensure timely diagnosis and prevent irreversible damage.

### 3.1 Discussion

The most frequent neurological manifestation of acquired copper deficiency is myelopathy or myeloneuropathy [4]. Patients usually present with a subacute gait disorder characterized by pronounced sensory ataxia and/or spasticity. Long tract signs, including spasticity and a positive Babinski response, are common findings in neurological examinations. Impaired vibration, position, and a positive Romberg sign are also common. Although bladder dysfunction is relatively rare, it can occur [4]. This neurological deficit pattern indicates the involvement of the dorsal columns of the spinal cord, resembling the deficits observed in patients with SCD of the spinal cord due to vitamin B12 deficiency. Our patient presented with numbness, tingling, and weakness of the lower extremities. He also had a positive Babinski response and loss of lower extremity position and vibration senses. Due to the similar clinical presentation of SCD and CDM, our patient was initially treated for SCD despite normal serum vitamin B12 levels. Because of this similar clinical presentation, there should be a higher index of suspicion for CDM as a differential diagnosis of SCD of the spinal cord, especially in individuals who do not respond to treatment.

Acquired copper deficiency is mainly caused by malabsorption and excess zinc intake. However, dietary copper insufficiency is rare. Among the causes, surgery is the most common [12]. Bariatric surgery, gastrectomy, and intestinal bypass surgery frequently cause copper deficiency [11, 12, 20, 22, 23, 24, 25]. Other causes include malabsorption syndromes, such as celiac disease, Wilson disease overtreatment, and total parenteral nutrition, which have also been reported to be linked to CDM [26, 27, 28, 29, 30]. Our patient did not have any of the aforementioned causes. The cause remains unknown in approximately 20% of the copper deficiency cases [4].

Myelopathy is a rare but potentially reversible neurological manifestation of copper deficiency. Reported cases of copper deficiency myeloneuropathy range in age from 30 to 82 years, with a higher prevalence in women than in men [11, 21]. Our patient's age also falls in this category. The serum level of copper tends to be very low or undetectable in cases of CDM [31, 32]; however, our patient had a marginally low copper level. Clinically and radiologically, it closely resembles SCD of the spinal cord, caused by vitamin B12 deficiency. This necessitates a high index of suspicion for copper deficiency, especially in cases with normal vitamin B12 levels [4, 33]. Copper deficiency often affects the posterior column and the lateral corticospinal tract of the spinal cord. It presents with sensory ataxia, spastic paraparesis, impaired vibration, and position sensation with or without distal sensory symptoms [33, 34]. Rarely, it can involve the brain stem and optic nerve [34, 35].

Copper deficiency can also manifest in other organ systems, such as in hematologic abnormalities. Hematologic manifestations of copper deficiency typically include anemia or neutropenia and, more rarely, pancytopenia [21]. Copper deficiency can negatively affect cholesterol and glucose metabolism [35]. Our patient showed no other organ manifestations.

The diagnosis of CDM is challenging. It requires a high index of suspicion because it is difficult to distinguish it from SCD. Laboratory indicators of copper deficiency include decreased serum copper and ceruloplasmin levels, with over 90% of the circulating copper bound to ceruloplasmin. Measuring 24‐h urinary copper excretion is a less sensitive marker for detecting copper deficiency [36]. All documented cases of copper deficiency myeloneuropathy have shown low serum copper levels [11, 31, 32]. However, serum copper levels may remain within the normal range in cases of marginal copper deficiency [37, 38].

Our patient's serum copper level was relatively higher than that in other CDM cases, indicating a probable marginal deficiency.

Vitamin B12 levels should be evaluated, as vitamin B12 deficiency may coexist with copper deficiency, especially in patients with a history of gastric surgery [11]. Measurements of methylmalonic acid and homocysteine levels, which are elevated during vitamin B12 deficiency, should be conducted in high‐risk patients. Serum vitamin B12 levels were normal in this patient, and methylmalonic acid levels were not determined for financial reasons, but CDM should have been suspected earlier.

In SCD, vitamin B12 supplementation typically improves neurological symptoms, whereas copper deficiency progression continues despite vitamin B12 treatment [12, 14, 23]. Vitamin B12 treatment was also attempted on our patient but with no improvement. Consistent clinical features and low serum copper or ceruloplasmin levels can confirm the diagnosis of CDM [14]. Other conditions that mimic CDM include nitrous oxide toxicity, cervical spondylotic myelopathy, small vessel vasculitis due to autoimmune diseases (systemic lupus erythematosus, Sjogren's disease, antiphospholipid antibody syndrome), multiple sclerosis, infe5ctious diseases (e.g., syphilis, HIV myelopathy, HTLV 1, herpes virus myelitis), other trace element deficiencies (e.g., vitamin E deficiency), and malignancies (e.g., astrocytoma, ependymoma, and epidural metastases) [39, 40, 41].

Neuroimaging with MRI should be performed for patients who present with signs and symptoms of myelopathy. No specific MRI findings can be used to diagnose CDM. Spinal MRI findings may be normal. A Mayo Clinic review of 25 patients with CDM found that 14 had normal spinal MRI. In another literature review of 55 case reports, only 47% were abnormal [21]. Imaging findings include T2 hyperintensities without contrast enhancement in the posterior and/or lateral column of the thoracic or cervical spinal cord [12, 42]. Our patient's spinal and brain MRI was normal.

Nerve conduction studies and other electrophysiologic tests are not crucial for diagnosing or evaluating patients with copper deficiency myeloneuropathy. Still, they may be used during the initial assessment of a patient's symptoms. In patients with copper deficiency myeloneuropathy, nerve conduction studies generally show an axonal sensorimotor polyneuropathy of varying severity [20, 43, 44]. Due to the unavailability of these tests, nerve conduction studies were not performed in our patients.

Copper supplementation and treatment of the underlying cause are the treatments for CDM. This approach can lead to stabilization or partial improvement of neurological deficits. No studies have determined the optimal dose, duration, route, or form of copper supplementation, though various regimens have been successfully employed. Commonly used copper salts include copper gluconate, copper sulfate, and copper chloride. Routine monitoring has been recommended in high‐risk populations [45]. Our patient was treated with cupric chloride 5 mg orally/day for 9 months.

In a study by Jaiser SR et al. examining 47 reported cases, 49% of patients experienced improvement, while 51% achieved stabilization [21]. Notably, none of the patients achieved full recovery. In contrast, our patient made a full recovery. Early intervention is crucial to prevent irreversible neurological damage. In comparison, treatment of SCD is also effective, but some patients achieve complete resolution of symptoms [46].

The absence of methylmalonic acid and homocysteine measurements is a limitation. These tests are more sensitive indicators of B12 deficiency than serum B12 alone and could have further strengthened the argument against a B12 deficiency diagnosis. The other limitation of this case is the unknown cause of copper deficiency. Although malabsorption was ruled out, the precise cause of the copper deficiency in this patient remains unclear. This makes it difficult to draw broader conclusions about the etiology of CDM.

In conclusion, this case report highlights the importance of considering CDM in the differential diagnosis of SCD, even when vitamin B12 levels are normal. The patient's initial presentation mimicked SCD, leading to delayed diagnosis of CDM. This underscores the need for a high index of clinical suspicion for CDM, especially in patients presenting with sensory ataxia, spasticity, and paresthesia, who do not respond to vitamin B12 therapy. This case also demonstrates the potential for complete recovery with oral copper supplementation in CDM, even after significant neurological impairment. The patient's full recovery emphasized the importance of early diagnosis and treatment to prevent irreversible neurological damage. Although this patient had a relatively higher serum copper level than that typically reported in CDM, it was still below the reference range, and the diagnosis was confirmed in conjunction with the clinical picture. This case suggests that marginal copper deficiency can lead to significant neurological symptoms.

## Author Contributions

**Zekarias Seifu Ayalew:** resources, validation, writing – original draft. **Mehariw Wondimu Netsere:** supervision, validation, writing – original draft. **Matyas Adugna Abebe:** resources, validation, writing – original draft. **Surafel Tilahun Maru:** resources, validation, writing – original draft. **Gebeyehu Tessema Azibte:** software, writing – review and editing. **Aynalem Demsis Biza:** visualization, writing – review and editing. **Getnet Yigzaw Mossie:** software, writing – review and editing.

## Ethics Statement

The authors have nothing to report.

## Consent

Written informed consent was obtained from the patient to publish this report in accordance with the journal's patient consent policy.

## Conflicts of Interest

The authors declare no conflicts of interest.

## Data Availability

The corresponding author has all the data and materials for this case report.
